# HP1γ function is required for male germ cell survival and spermatogenesis

**DOI:** 10.1186/1756-8935-3-9

**Published:** 2010-04-27

**Authors:** Jeremy P Brown, Jörn Bullwinkel, Bettina Baron-Lühr, Mustafa Billur, Philipp Schneider, Heinz Winking, Prim B Singh

**Affiliations:** 1 Division of Immunoepigenetics, Department of Immunology and Cell Biology, Research Center Borstel, D-23845 Borstel, Germany; 2 Institut für Biologie, Universität Lübeck, D-23538 Lübeck, Germany

## Abstract

**Background:**

HP1 proteins are conserved components of eukaryotic constitutive heterochromatin. In mammals, there are three genes that encode HP1-like proteins, termed HP1α, HP1β and HP1γ, which have a high degree of homology This paper describes for the first time, to our knowledge, the physiological function of HP1γ using a gene-targeted mouse.

**Results:**

While targeting the *Cbx3 *gene (encoding the HP1γ protein) with a conditional targeting vector, we generated a hypomorphic allele (*Cbx3*^*hypo*^), which resulted in much reduced (barely detectable) levels of HP1γ protein. Homozygotes for the hypomorphic allele (*Cbx3*^*hypo*/*hypo*^) are rare, with only 1% of *Cbx3*^*hypo*/*hypo *^animals reaching adulthood. Adult males exhibit a severe hypogonadism that is associated with a loss of germ cells, with some seminiferous tubules retaining only the supporting Sertoli cells (Sertoli cell-only phenotype). The percentage of seminiferous tubules that are positive for L1 ORF1 protein (ORF1p) in *Cbx3*^*hypo*/*hypo *^testes is greater than that for wild-type testes, indicating that L1 retrotransposon silencing is reversed, leading to ectopic expression of ORF1p in *Cbx3*^*hypo*/*hypo *^germ cells.

**Conclusions:**

The *Cbx3 *gene product (the HP1γ protein) has a non-redundant function during spermatogenesis that cannot be compensated for by the other two HP1 isotypes. The *Cbx3*^*hypo*/*hypo *^spermatogenesis defect is similar to that found in *Miwi2 *and *Dnmt3L *mutants. The *Cbx3 *gene-targeted mice generated in this study provide an appropriate model for the study of HP1γ in transposon silencing and parental imprinting.

## Background

The presence of methylated lysine 9 of histone H3 (H3K9ME) and structural heterochromatin protein (HP) 1 proteins are characteristic evolutionarily conserved hallmarks of heterochromatin [1]. In mammals, there are three HP1 isotypes, which have a high degree of homology, termed HP1α (encoded by the *Cbx5 *gene), HP1β (encoded by the *Cbx1 *gene) and HP1γ (encoded by the *Cbx3 *gene) [2,3]. Despite the significant degree of sequence conservation shared between the mammalian HP1 isotypes, several studies have indicated that they are likely to have non-redundant functions. First, their nuclear localization patterns are different: HP1α and HP1β are usually found enriched at sites of constitutive heterochromatin, whereas HP1γ has a more uniform distribution [4-6]. Second, biochemical assays have identified isotype-specific binding partners [7] and, third, targeted deletion of the *Cbx1 *gene has shown that it is essential, and that its loss of function cannot be compensated for by the products of the *Cbx5 *and *Cbx3 *genes [8].

Analysis of the *Cbx1 *null mutant has shown that the *Cbx1 *gene product, HP1β, is required for proper development of the brain, with *Cbx1*^-/- ^neurospheres cultured *in vitro *showing a dramatic genomic instability that is indicative of a defect in centromere function [8]. Interestingly, the lethality of the *Cbx1 *mutation compared with the observed viability of the *Suv(3)9h1*/*h2 *histone methyl transferase (HMTase) double mutant [9] shows that the essential function(s) of HP1β lies outside its interaction with the heterochromatic H3K9ME3 determinant generated by the Suv(3)9h1/h2 HMTases [3]. By contrast, homozygous *Cbx5*^-/- ^mutants are indistinguishable from wild-type littermates, indicating that its function is redundant [8] (Singh PB: unpublished data). To date, nothing is known about the biological function of the *Cbx3 *gene.

The mouse *Cbx3 *gene lies on chromosome 6, and is tightly linked to the *Hnrnpa2b1 *gene [10]. Both genes, which are divergently transcribed, share a 3 kb CpG island that is conserved in the syntenic *HNRNPA2B1*-*CBX3 *region in humans [11]. Fragments from the CpG-rich *HNRNPA2B1*-*CBX3 *region have been shown to be able to confer high-level expression of linked transgenes in the mouse and thus, it has been termed a ubiquitously acting chromatin opening element (UCOE) [12,13].

In a first attempt to elucidate the biological function of the *Cbx3 *gene, we undertook a gene-targeting experiment using a conditional targeting vector. During production of this conditional mutation, we fortuitously generated a hypomorphic allele of *Cbx3 *(*Cbx3*^*hypo*^), which results in a dramatic reduction in HP1γ protein expression to barely detectable levels; expression of the Hnrnpa2b1 protein was not affected. The number of *Cbx3*^*hypo*/*hypo *^homozygotes that survive to adulthood is low, with adult males exhibiting a severe spermatogenic defect. This result confirms the non-redundant functions of mammalian HP1 proteins, and provides the first insight into the function of HP1γ during development. We also observed a dramatic reduction in the number of germ cells in *Cbx3*^*hypo*/*hypo*^, with a concomitant increase in expression of the ORF1 protein encoded by the LINE-1 (L1) retrotransposon. These data indicate that HP1γ might be part of a Miwi2-HP1γ silencing pathway that is required for proper germ-cell renewal and survival in the testes.

## Results and discussion

**Figure 1:**
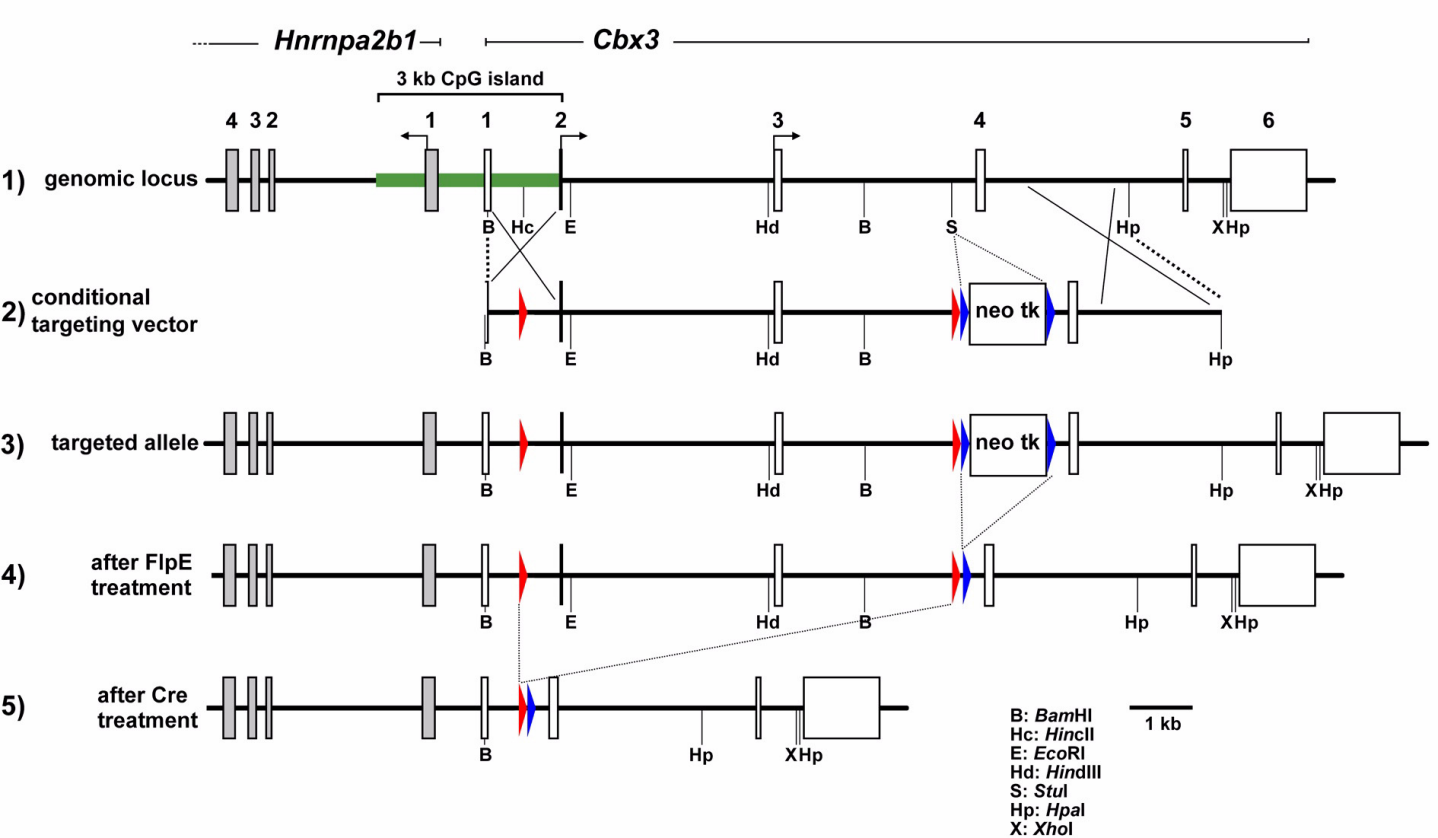
**Targeting of the *Cbx3 *locus with conditional vector**. Row 1, *hnrnpA2B1*/*Cbx3 *genomic locus in the mouse. The *hnrnpA2B1 *and *Cbx3 *genes are divergently transcribed and share a 3 kb CpG island (green). The ATG start sites are indicated by bent arrows; the alternative start sites for the *Cbx3 *gene are in exons 2 and 3. Row 2, conditional targeting vector. The *neo-tk *cassette is flanked by FRT sites (depicted as thin blue arrowheads) that can be used to excise the cassette by FlpE expression. The *Cbx3 *gene can be disrupted by *Cre *expression, which excises exons 2 and 3, containing the alternative ATG start sites. Row 3, *Cbx3 *genomic locus after targeting with the conditional vector shown in row 2, and represents the *Cbx3*^*hypo *^allele. Row 4, genomic locus after *FlpE *expression and removal of the *neo-tk *cassette. Row 5, genomic locus after *Cre *expression and excision of exons 2 and 3, giving rise to the *Cbx3*^-/-^null allele.

The conditional targeting vector used to disrupt the *Cbx3 *gene was designed with a FlpE-excisable *neo-tk *cassette placed in the intron separating exons 3 and 4 (Figure 1, second and third rows). Disruption of *Cbx3 *gene function, after excision of the *neo-tk *by FlpE, would be effected by *Cre *excision of exons 2 and 3, which contain the alternative ATG start codons of the *Cbx3 *gene (Figure 1, third and fourth rows). Targeting frequency with the *neo-tk*-containing conditional vector (Figure 1, second row) was approximately 1:100. Germline transmission of the targeted allele (Figure 1, third row) was achieved, and intercrossing of the heterozygotes for the targeted allele resulted in a normal mendelian ratio of wild-type to targeted alleles at E19 (not shown) the day before birth. However, the number of adult mice that were homozygous for the targeted allele was low, with only 3 of 216 adult males and 1 of 193 adult females being homozygous for the targeted allele (Figure 1, third row), which was designated *Cbx3*^*hypo*^. The nature and timing of the attrition of *Cbx3*^*hypo*/*hypo *^homozygotes that takes place after birth is not known, and the data presented below on *Cbx3*^*hypo*/*hypo *^adult animals are based on the three males and one female that reached adulthood.

**Figure 2:**
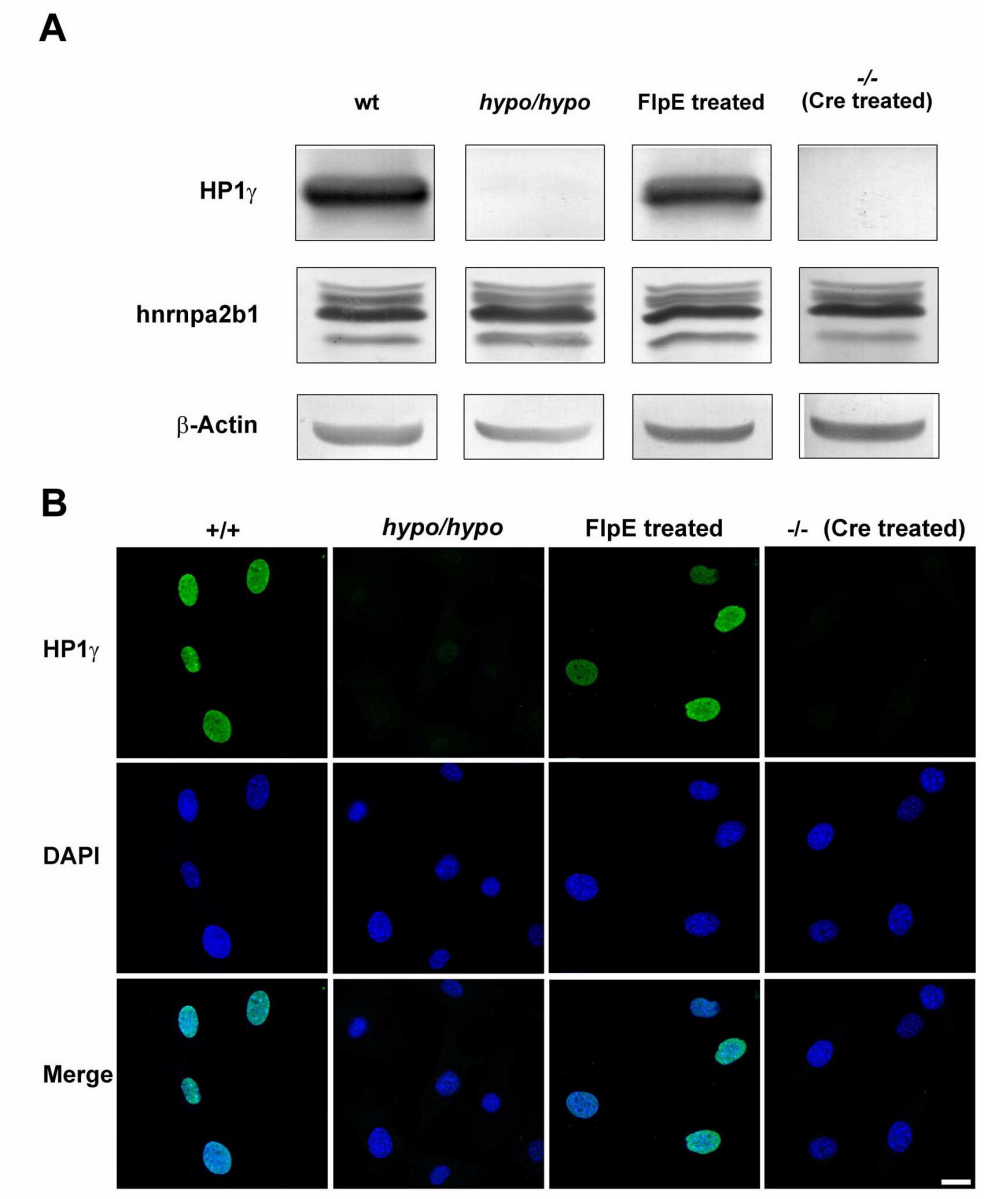
**Presence of the *neo-tk *cassette results in a hypomorphic (*Cbx3*^*hypo*^) allele**. **(a) **Row 1, expression of HP1γ in MEFs. HP1γ expression was dramatically reduced to almost undetectable levels in MEFs derived from embryos that are homozygous for the targeted allele shown in row 3 of Figure 1 (*Cbx3*^*hypo*/*hypo *^MEFs). After expression of FlpE in the *Cbx3*^*hypo*/*hypo *^MEFs (FlpE-treated MEFs), the expression of HP1γ returned to wild-type levels. HP1γ protein expression in the FlpE-treated MEFs could be extinguished by expression of *Cre *recombinase, giving rise to *Cbx3*^-/- ^MEFs. Hnrnpa2b1 protein expression was not affected by the presence or absence of the *neo-tk *cassette, as shown in row 2. Row 3, actin loading control. **(b) **Row 1, immunofluorescent HP1γ staining of MEFs. The typical punctate HP1γ pattern of expression in wild-type MEFs was reduced to very faint, almost background, levels in *Cbx3*^*hypo*/*hypo *^MEFs. Strikingly, HP1γ staining levels returned to wild-type levels after the *neo-tk *cassette was excised after Flpe treatment (FlpE-treated MEFs). HP1γ staining was lost after expression of *Cre *recombinase giving rise to *Cbx3*^-/- ^MEFs. Row 2, DAPI staining; row 3, merged images. Bar = 20 μm.

The greatly reduced numbers of *Cbx3*^*hypo*/*hypo *^adults prompted us to investigate whether the targeting event itself had affected *Cbx3 *expression, and if this was the likely cause of the reduced numbers of *Cbx3*^*hypo*/*hypo *^adults. To explore this hypothesis further, we generated primary mouse fibroblasts from E13.5 wild-type and *Cbx3*^*hypo*/*hypo *^littermates and compared the expression levels of HP1γ by Western blotting. As shown in Figure 2, there was a dramatic reduction in HP1γ expression levels in *Cbx3*^*hypo*/*hypo *^compared with wild-type mouse embryonic fibroblasts (MEFs) (Figure 2a, top row: wild-type to *hypo*/*hypo*) indicating that the *Cbx3*^*hypo *^allele was a hypomorph. The effect of the targeting event was specific to the *Cbx3 *gene, as protein expression of the closely linked *Hnrnpa2b1 *gene was not changed (Figure 2a, middle row). Given this unexpected result, we were prompted to investigate whether the presence of the *neo-tk *selection cassette itself was interfering with *Cbx3 *expression. Previous work has shown that knockdown of target gene expression can result from the presence of a *neo *gene in the targeting vector [14]. The mechanism for such a knockdown is not fully understood but may involve transcriptional interference, by which the presence of one transcriptional unit interferes with another that is in *cis *[15].

To test the hypothesis that the *neo-tk *selection cassette was interfering with *Cbx3 *expression, we took advantage of the fact that *neo-tk *cassette is flanked by FRT sites that allow its excision by FlpE expression (Figure 1). When the *neo-tk *cassette was excised after electroporation of FlpE mRNA into *Cbx3*^*hypo*/*hypo *^MEFs, the HP1γ expression levels returned to normal wild-type levels (Figure 2a, top row: FlpE treated) indicating that it was indeed the presence of the *neo-tk *cassette that resulted in the reduced HP1γ levels. As a control, the *Cbx3 *gene was disrupted by *Cre *expression, resulting in *Cbx3*^-/- ^MEFs and complete loss of HP1γ expression (Figure 2, top row: -/-). The Western blot analysis was complemented with immunofluorescence experiments, which confirmed that the presence of the *neo-tk *cassette affected *Cbx3 *expression (Figure 2b). The reduced levels of HP1γ protein in *Cbx3*^*hypo*/*hypo *^MEFs did not affect the expression of HP1α and HP1β as measured by immunofluorescence and Western blot analysis (see Additional files 1 and 2, Figures s1 and s2). Western blot analysis also revealed that there were no significant changes between *Cbx3*^*hypo*/*hypo *^and wild-type MEFs in the levels of three different histone post-translational modifications, H3K9ME3, H4K20ME3 and H3K9AC (see Additional file 2, Figure s2). When *Cbx3*^-/- ^MEFs were included into this analysis, we observed an increase in H4K20me3 levels compared with wild-type and *Cbx3*^*hypo*/*hypo *^MEFs (see Additional file 2, Figure s2), indicating that complete loss of HP1γ in *Cbx3*^-/-^cells might affect the activity of enzymes involved in regulating this determinant of the histone code.

**Figure 3:**
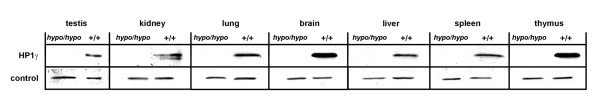
**HP1γ protein expression was dramatically reduced in *Cbx3*^*hypo*/*hypo *^tissues**. Protein expression was reduced to almost undetectable levels in testis, kidney, lung, brain, liver spleen and thymus tissues from the *Cbx3*^*hypo*/*hypo*^mice.

A dramatic reduction of HP1γ levels was also observed in the *Cbx3*^*hypo*/*hypo *^mouse tissues; Western blot analysis revealed that HP1γ levels were reduced to almost undetectable levels in all tissues examined (Figure 3). There was no effect of the *Cbx3*^*hypo*/*hypo *^mutation on the protein levels of HP1α (see Additional file 3, Figure s3a) and HP1β (see Additional file 3, Figure s3b) or on the same three histone post-translational modifications H4K20ME3, H3K9ME3 and H3K9AC (see Additional file 3, Figures s3c to s3e).

**Figure 4:**
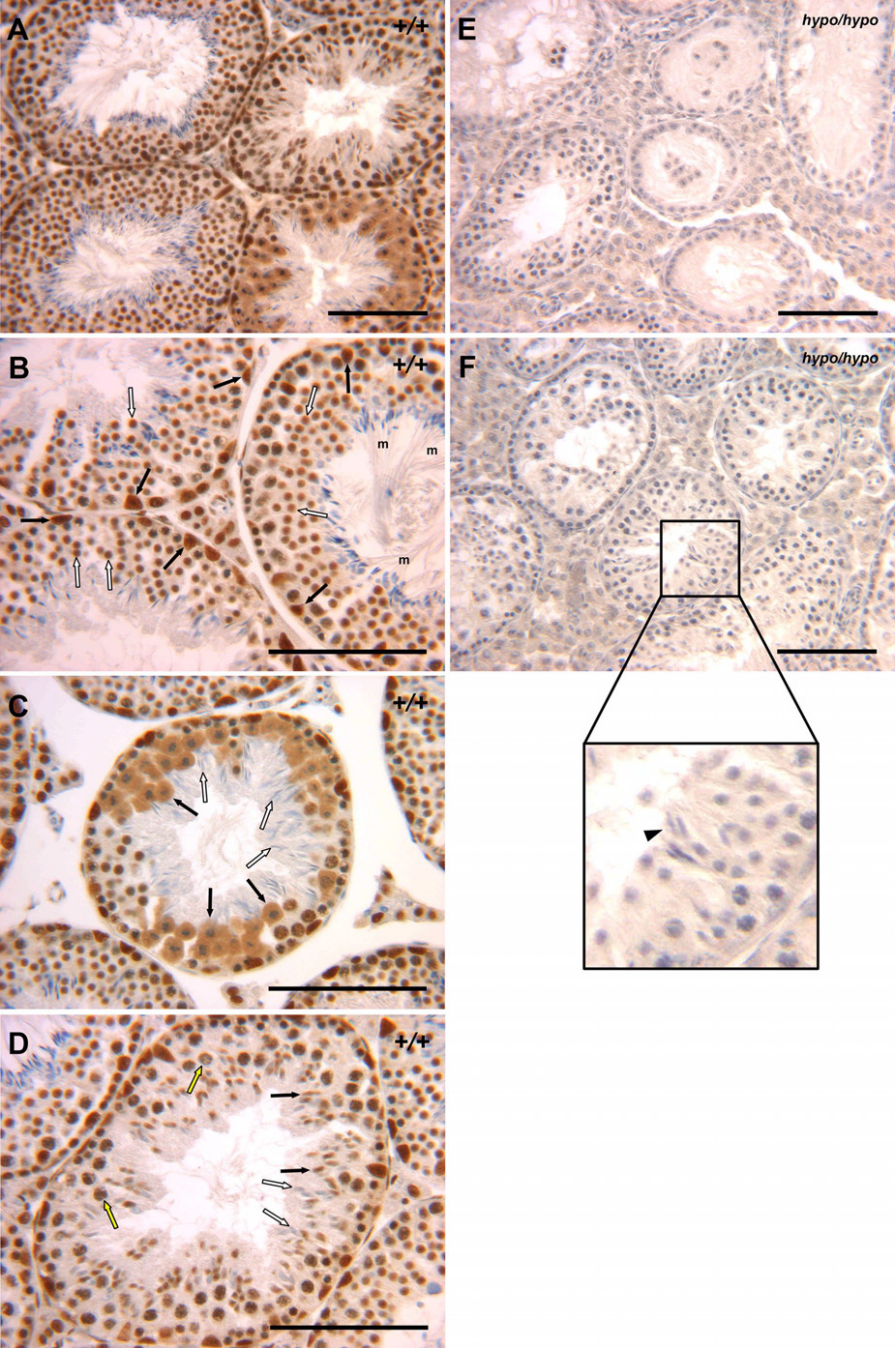
***Cbx3*^*hypo*/*hypo *^testes had severe impairment of spermatogenesis**. **(a) **HP1γ protein was found in nearly all cells of the wild-type testes. **(b) **In wild-type tubules, Sertoli cells stained strongly for HP1γ (black arrows) as did round spermatids (stage 2-6) (white arrows). Many round spermatid nuclei possessed an enriched focus of HP1γ staining that was characteristic of the single block of heterochromatin observed at this stage (see Additional file 5, Figures 5b). **(c) **HP1γ was largely excluded from meiotic metaphase chromosomes and was found surrounding the condensed chromosomes in the meiotic cytoplasm (black arrows). Mature sperm (white arrows) were negative for HP1γ. **(d) **Stage 10 spermatids, at around the time they elongated, were either positive (black arrows) or negative (white arrows) for HP1γ, indicating that it was during this stage that levels of HP1γ proteins decrease. Pachytene spermatocytes (yellow arrows) showed a few brightly stained spots that represent sites of constitutive heterochromatin (see Additional file 5, Figures 5a). **(e) **In *Cbx3*^*hypo*/*hypo *^testes, HP1γ staining was very weak and the tubules showed severely impaired spermatogenesis with greatly reduced numbers of cells and some tubules exhibiting a Sertoli cells-only phenotype (see upper right tubule). **(f) **In some tubules of *Cbx3*^*hypo*/*hypo *^testes, mature sperm could be observed (inset, black arrowhead). Bar = 100 μm.

Housing the three *Cbx3*^*hypo*/*hypo *^males with wild-type females resulted in no litters, indicating a possible problem with male fertility. The males were killed and the testes removed, which revealed severe hypogonadism compared with wild-type males (Additional file 4, Figure s4). Examination of wild-type testes showed that expression of the *Cbx3 *gene product, HP1γ, was present in virtually all cells (Figure 4a) and is distinct from the staining pattern of transcriptional intermediary factor (TIF)1β, a HP1γ-interacting protein, in wild-type testes, where pre-leptotene and spermatogonia are TIF1β-negative [16]. Sertoli cells are prominently stained, as are round (stage 2-6) spermatids (Figure 4b), with the latter showing an enriched spot of HP1γ staining in the nucleus (Figure 4b; see also Additional file 5, Figure s5b), which probably represents the characteristic heterochromatic chromocenter found in these nuclei [17]. HP1γ is excluded from meiotic metaphase chromosomes (Figure 4c), which is similar to the known expulsion of the bulk of HP1 proteins from metaphase chromosomes by the so-called serine 10 phosphorylation switch [18]. It may well be that the chromosome condensation during meiosis or mitosis requires the removal of the bulk of HP1 proteins from the chromosomes. In pachytene spermatocytes, HP1γ is found throughout the nucleus but is enriched at a few sites that probably represent heterochromatic chromocenters (Figures 4d; see also Additional file 5, figure s5a). HP1γ staining of spermatogenic cell types is detectable until elongating spermatid stage 10 (Figure 4d; Table 1), which is around the time that the bulk of the histones are removed and replaced by the protamines [19].

**Table 1:** Spermatid stages at which HP1α, HP1β and HP1γ protein expression was extinguished.

Protein	Spermatid stage number				
	
	7	8	9	10	11
HP1α	+	-	-	-	-

HP1β	+	+	+	+	-

HP1γ	+	+	+	+	-

**Figure 5:**
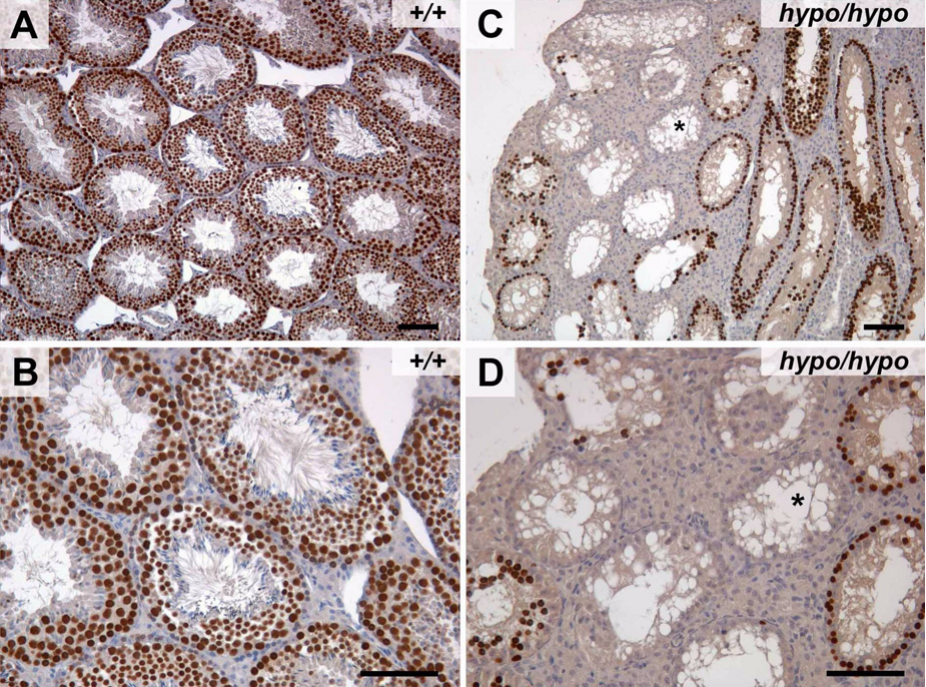
***Cbx3*^*hypo*/*hypo *^testes showed a dramatic loss in the number of germ cells**. **(a,b) **Typical germ cell nuclear antigen (GCNA) staining of germ cells within wild-type testes. **(c,d) **In *Cbx3*^*hypo*/*hypo *^testes, there was a dramatic reduction in the numbers of GCNA-positive germ cells. Some tubules contained no GCNA-positive germ cells and presented a Sertolin cells-only phenotype (asterisk). Bar = 100 μm.

By contrast, HP1γ staining was almost undetectable in sections taken from *Cbx3*^*hypo*/*hypo *^testes (Figure 4e, f). Histological examination revealed a severe impairment of spermatogenesis in *Cbx3*^*hypo*/*hypo *^testes. The diameter of the tubules in *Cbx3*^*hypo*/*hypo *^testes was smaller (0.13 mm) than that in wild-type testes (0.2 mm). Of 70 tubules examined, 22 were almost completely devoid of germ cells (for example, Figure 4e, upper right tubule) and 48 tubules had impaired spermatogenesis. Tubules in which mature sperm could be observed were rare (Figure 4f, especially the magnified inset). The loss of germ cells was confirmed using a germ-specific antibody (anti-germ cell nuclear antigen (GCNA)) [20], which revealed a dramatic reduction in germ cells (GCNA-positive cells) in *Cbx3*^*hypo*/*hypo *^testes (Figure 5c, Figure 5d) compared with wild-type mice (Figure 5, Figure 5b). Some of the tubules in *Cbx3*^*hypo*/*hypo *^testes exhibited a Sertoli cell-only (SCO) phenotype, reminiscent of the tubules seen in the *Dnmt3L *and *Miwi2 *mutants [21,22]. Thus, the *Cbx3*^*hypo*/*hypo *^mutation results in the general suppression of spermatogenesis, which can vary from tubule to tubule; in some tubules suppression is complete, resulting in a SCO phenotype, whereas in others, spermatogenesis can proceed and give rise to some mature sperms. This variation across the tubules might reflect the fact that the *Cbx3*^*hypo *^mutation is 'leaky', that is, the interference of *Cbx3 *by the *neo-tk *is variable giving rise to leaky expression of *Cbx3*. Some *Cbx3*^*hypo*/*hypo *^germ cells and their progeny might have closer to wild-type levels of HP1γ, thus enabling greater likelihood of survival with more normal development.

The similarity of the *Cbx3*^*hypo*/*hypo *^spermatogenesis defect to *Dnmt3L *and *Miwi2 *mutants [21,22] prompted us to investigate whether there were any changes in the expression of retrotransposon expression in the mutant testes. For this, we used a polyclonal antibody to the L1-encoded ORF1 protein [23]. ORF1p is required for L1 transposition, and its levels of expression are increased in germ cells, as the L1 transposons become de-repressed [24,25]. Using this antibody, we found that 45% of the tubules in *Cbx3*^*hypo*/*hypo*^testes that contained germ cells were positive by immunohistochemistry for ORF1 protein expression, compared with 5% in wild-type testes (see Additional file 6 and 7, Figure s6 and Figure s7). Again, this indicates that the *Cbx3*^*hypo*/*hypo *^mutation may affect the same silencing pathway that is affected in the *Dnmt3L *and *Miwi2 *mutants [21,22].

We next investigated whether the *Cbx3*^*hypo *^mutation affects the expression of the other two HP1 isotypes, HP1α and HP1β. Accordingly, we stained wild-type and *Cbx3*^*hypo*/*hypo *^testes with HP1α and HP1β antibodies, and compared the cell types and levels of staining for the two proteins on the different genetic backgrounds. For HP1α, most cells of the wild-type testes were HP1α-positive (see Additional file 8, Figure s8). Sertoli cells were stained with anti-HP1α (see Additional file 8, Figure s8, black arrows) as were pachytene spermatocytes, where HP1α was enriched within a few bright foci which probably represent heterochromatic regions (see Additional file 8, Figure s8b, blue arrows). The round (stage 2-6) spermatids were also stained and exhibited a single spot of staining in the nucleus, which is characteristic of the heterochromatic chromocenter found in these cell types (see Additional file 8, Figure s8c, white arrows). Meiotic chromosomes were not stained but, unlike HP1γ staining in wild-type testes, very little staining was observed in the meiotic cytoplasm (see Additional file 8, Figure s8c, arrowheads). In addition, unlike with HP1γ, there are some cells, probably spermatogonia, which were not stained by the anti-HP1α antibody (see in Additional file 8, Figure s8b, yellow arrows). In wild-type testes, HP1α staining was lost at an earlier stage than HP1γ staining, with stage 7 spermatids (see Additional file 8, Figure s8b, arrowheads) being the last stage at which HP1α was still seen (Table 1). In the *Cbx3*^*hypo*/*hypo *^testes, the cell types stained were the same as those found in wild-type cells, notwithstanding the obvious suppression of spermatogenesis seen in the *Cbx3*^*hypo*/*hypo *^testes (see Additional file 8, Figure s8d, Figure s8e). The levels of HP1α staining were also unchanged in the *Cbx3*^*hypo*/*hypo *^testes, as evidenced by the typical staining of the round (stage 2-6) spermatids in *Cbx3*^*hypo*/*hypo *^testes (see Additional file 8, Figure s8e, white arrows). HP1β staining of wild-type testes (see Additional file 9, Figure s9) was similar to that for HP1α (see Additional file 8, Figure s8), with the only difference being that the staining of HP1β was still visible at a later stage, in stage 10 spermatids, as was seen for HP1γ (Table 1) [26]. The levels of HP1β staining and the cell types stained in *Cbx3*^*hypo*/*hypo *^testes (see Additional file 9, Figure s9d, Figure s9e) were not significantly different to those in the wild-type testes. These data indicate that the defects seen in the *Cbx3*^*hypo*/*hypo *^mutation are unlikely to operate through changes in the expression of the other two isotypes, HP1α and HP1β.

The clear differences between wild-type and *Cbx3*^*hypo*/*hypo*^adult testes were not observed in embryonic E17 testes. The morphology of the seminiferous tubules and numbers of gonocytes in wild-type and *Cbx3*^*hypo*/*hypo *^testes were similar (see Additional file 10, Figure s10), indicating that the suppression of spermatogenesis seen in the adult *Cbx3*^*hypo*/*hypo *^testes (Figure 4) probably occur at later stages, after meiosis has been initiated.

Housing the one adult *Cbx3*^*hypo*/*hypo *^female mouse with wild-type males also resulted in no litters. Although it is difficult to conclude from a single *Cbx3*^*hypo*/*hypo *^animal that *Cbx3*^*hypo*/*hypo *^females are sterile, we nevertheless decided to examine sections of wild-type and *Cbx3*^*hypo*/*hypo *^ovaries. Examination of the sections revealed no obvious morphological difference between wild-type and *Cbx3*^*hypo*/*hypo *^ovaries; all stages of folliculogenesis were observed in *Cbx3*^*hypo*/*hypo *^ovaries, including corpora lutea, indicating that ovulation was normal in the *Cbx3*^*hypo*/*hypo *^female (data not shown).

The similarity of the *Cbx3*^*hypo*/*hypo *^phenotype in the testes with those observed in the testes of *Miwi2 *[21] and *Dnmt3L *mutants [21] is suggestive. Both *Miwi2 *and *Dnmt3L *are involved in DNA methylation of interspersed repeats during spermatogenesis, and mutation of either *Miwi2 *or *Dnmt3L *results in a SCO phenotype and the loss of DNA methylation of transposons, resulting in their ectopic expression [21,22]. Our analysis of the *Cbx3*^*hypo*^mutation indicates that HP1γ might also be involved in a Miwi2-HP1 silencing pathway, as observed for HP1a-PIWI pathway in *Drosophila *[27]. These data, in conjunction with the generation of a *Cre*-inducible *Cbx3 *allele from the *Cbx3*^*hypo *^allele (unpublished), form a sound basis for investigating the role of HP1γ in transposon silencing and parental imprinting.

## Conclusion

HP1γ has a non-redundant function that cannot be rescued by the other HP1 isotypes, HP1α and HP1β. This function is essential for male germ cell survival and proper spermatogenesis.

## Methods

### Animal studies

The experimental research on mice was carried out in accordance with German animal protection law, and the study has been approved by the Ministerium für Landwirtschaft, Umwelt und ländliche Räume of Schleswig-Holstein in Kiel (Germany).

### Staining of testes sections

Testes were fixed in Bouin's fixative (saturated aqueous solution of picric acid, 37% formaldehyde, and glacial acetic acid, 15:5:1) overnight, embedded in paraffin wax and cut into sections 2 μm thick. Subsequent antigen retrieval by pressure cooker and indirect immunoperoxidase staining was performed as described previously [28]. In addition, blocking solution (Image-iT FX Signal Enhancer; Invitrogen, Carlsbad, CA, USA) was applied for 30 minutes to reduce background staining. All antibodies were diluted in Tris-buffered saline with 10% bovine serum albumin (BSA). Endogenous peroxidase was inactivated with 3% H_2_O_2_, and diaminobenzidine (DAB; Sigma, St. Louis, MO, USA) was used to detect peroxidase activity. Primary antibodies used in this study were anti-GCNA1 (mAB 10D9G11, kind gift of Professor G C Enders), anti-HP1α, anti-HP1β [4] and anti-HP1γ (all Chemicon, Temecula, CA, USA). Species-specific horseradish-peroxidase coupled secondary antibodies were purchased from Dianova (Hamburg, Germany). Images were photographed with a microscope and camera (DMLB2 microscope and DFC320 camera; Leica, Basel, Switzerland).

For L1 ORF1p staining, paraffin wax-embedded sections were dewaxed and subsequently incubated for 15 minutes with 1% peroxide followed by 15 minutes with signal enhancer (Image-iT FX Signal Enhancer; Invitrogen). For antigen retrieval, pancreatic trypsin (1 mg/ml in phosphate-buffered saline (PBS)) was applied for 2 minutes. The samples were incubated with anti-mouse ORF1p antibody (kind gift of Professor S L Martin) at 1:500 dilution in PBS with 10% BSA overnight at 4°C, with secondary antibody and peroxidase detection with DAB performed as described above. Finally, the sections were incubated with haematoxylin for 6 minutes. Images were taken with a Olympus (Hamburg, Germany) DS-Ri1 microscope, an Nikon (Melville, NY, USA) BX41 camera and NIS-Elements documentation software. Tubules were scored negative for ORF1p if the resident germ cells exhibited haematoxylin staining only with no brown staining (see Additional file 6, Figure s6c, Figure s6e). Tubules that were scored positive for ORF1p contained germ cells with robust brown staining of the nucleus and cytoplasm (see Additional file 6, Figure 6c, Figure s6e). *Cbx3*^*hypo*/*hypo *^tubules that had no germ cells (see Additional file 6, Figure s6b, asterisks) were not included in the analysis.

### Western blotting

Western blotting was performed essentially as described previously [8]. For histone isolation, tissues were cut to pieces and further disintegrated in buffer A (10 mM HEPES, pH 7.9, 1.5 mM MgCl_2_, 10 mM KCl, 0.5 mM dithiothreitol (DTT) and Complete Mini EDTA-free Protease Inhibitor (Roche, Mannheim, Germany)) with a Dounce homogenizer (50 strokes with a tight pestle). Nuclei were pelleted and then resuspended in buffer S1 (0.25 M sucrose, 10 mM MgCl_2 _and protease inhibitor) and layered over an equal volume of buffer S3 (0.88 M saccharose, 0.5 mM MgCl_2 _and protease inhibitor). After separation by centrifugation (2,800 ***g ***for 10 minutes at 4°C) the pellet was resuspended in extraction buffer (1 M HCl, 0.02% β-βmercaptoethanol and protease inhibitor) and incubated at 4°C overnight. Pellet was extracted twice. The supernatants were pooled and treated with 10 volumes of acetone for precipitation (- 20°C overnight). After separation by centrifugation (10,000 g ***g ***for 4 minutes at 4°C), the pellet was reconstituted in water and finally denatured in Laemmli buffer (5 minutes at 95°C) for SDS-PAGE.

For Western blotting, primary antibodies for HP1α, HP1β, HP1γ, histone 3, histone 4, H3K9AC (all Chemicon), β-Actin (Sigma), hnRNP A2B1 (Biozol, Eching, Germany), H3K9ME3 [29] and H4K20ME3 [30] were used. Detection of these antibodies was carried out either with colorimetric reaction through species-specific AP-coupled secondary antibodies (Dianova, Germany) or by incubation with infrared dye-coupled secondary antibodies (Alexa Fluor 680-coupled goat anti-mouse (Invitrogen) and IRDye 800 CW anti-rabbit (LI-COR, Lincoln, NE, USA)) and subsequent scanning with an infrared imager (Odyssey; LI-COR).

### Immunofluorescence

Immunofluorescence was performed as described previously [31]. Primary antibodies used in this study were against HP1α, HP1β and HP1γ (all Chemicon). Nucleic acids were stained with 4',6-diamidino-2-phenylindole (1 μg/ml). Confocal images were acquired, processed and assembled as described previously [8].

## Competing interests

The authors declare that they have no competing interests.

## Authors' contributions

JPB made the conditional targeting vector and targeted ES cells, and helped to analyse the data. JB assembled all figures for manuscript and helped to analyse the data. MB and PS undertook staining of cells and Western blotting. BB-L undertook all the immunocytochemistry on testes sections. HW analysed the staining of testes. PBS conceived of and designed the study, and wrote the first draft of the paper.

## Supplementary Material

Additional file 1 **Figure s1**. Immunofluorescent staining of HP1α and HP1β was not changed in wild-type and *Cbx3 *mutant backgrounds. **(a) **There was no significant difference in HP1α staining on wild-type and *Cbx3 *mutant backgrounds (row 1, green). Row 2, staining with 4',6-diamidino-2-phenylindole (DAPI) of cells depicted in the panels above. Images of rows 1 and 2 were merged in row 3. **(b) **There was no significant difference in HP1β staining on wild-type and *Cbx3 *mutant backgrounds (row 1, green). Row 2, DAPI staining of cells depicted in the panels above. Images of rows 1 and 2 were merged in row 3. Bar = 20 μm. Click here for file

Additional file 2 **Figure s2**. There was no significant difference in the levels of HP1α, HP1β, H3K9ME3, H3K9AC in wild-type MEFs compared with *Cbx3 *mutant MEFs. There was a slight increase in H4K20ME3 levels in *Cbx3*^-/- ^MEFs compared with wild-type and *Cbx3*^*hypo*/*hypo *^MEFs. Click here for file

Additional file 3 **Figure s3**. There was no significant difference in the levels of HP1α, HP1β, H3K9ME3, H4K20ME3, H3K9AC in *Cbx3*^*hypo*/*hypo *^extracts taken from testis, kidney, lung, brain, liver, spleen and thymus tissues compared with extracts for the corresponding wild-type tissues. ND = not detectable. Click here for file

Additional file 4 **Figure s4**. Testes from *Cbx3*^*hypo*/*hypo *^males exhibited severe hypogonodism. Bar = 50 mm. Click here for file

Additional file 5 **Figure s5**. **(a) **Many pachytene spermatocytes (black arrows) from wild-type animals had punctuate staining, with the intensely staining areas probably representing regions of heterochromatin (see inset). **(b) **Many round spermatids from wild-type animals exhibited a 'spot' that was enriched for HP1γ staining and probably represents the single block of heterochromatin that was characteristically found in these cells. Click here for file

Additional file 6 **Figure s6**. LINE-1 (L1) retrotransposon expression was increased in *Cbx3*^*hypo*/*hypo *^compared with wild-type testes, as shown by an increase in germ cells staining positive for the L1-encoded ORF1 protein (ORF1p). **(a) **A wild-type testis section stained with the anti-L1-encoded ORF1p antibody, showing regions from two tubules, one tubule negative for ORF1p and the other tubule positive for ORF1p, which are magnified in **(c) **and **(d)**, respectively. **(b) **A *Cbx3*^*hypo*/*hypo *^testis section stained with the anti-L1-encoded ORF1p antibody, showing regions from two tubules, one tubule negative for ORF1p and the other tubule positive for ORF1p, which are magnified in **(e) **and **(f)**, respectively. The asterisks denote tubules that have the Sertoli cells-only phenotype and lack germ cells. **(c) **ORF1p-negative germ cells in a wild-type tubule. **(d) **ORF1p-positive germ cells in a wild-type tubule. **(e) **ORF1p-negative germ cells in a *Cbx3*^*hypo*/*hypo *^tubule. **(f) **ORF1p-positive germ cells in a *Cbx3*^*hypo*/*hypo*^tubule. Bar = 200 μm. Click here for file

Additional file 7 **Figure s7**. In the *Cbx3*^*hypo*/*hypo *^testes section, 45% of the tubules contain ORF1p-positive germ cells compared with 5% of the tubules in the wild-type testes section. Click here for file

Additional file 8 **Figure s8**. Distribution and levels of HP1α staining in testes was not affected by the *Cbx3*^*hypo*/*hypo *^mutation. **(a) **Most cells within the wild-type testes were stained with HP1α antibody. **(b) **A more detailed analysis of the HP1α staining showed that Sertoli cells (black arrows) were clearly stained. Pachytene spermatocytes (blue arrows) exhibited punctuate staining, probably representing heterochromatin. HP1α staining was lost in stage 7 round spermatids (black arrowhead), which was earlier than for HP1γ (see Figure 4; Table 1). In contrast to the HP1γ staining, there were some cells at the periphery of the tubule, probably spermatogonia (yellow arrows), which were not stained. **(c) **HP1α does not stain meiotic chromosomes (black arrowheads) and was present at low levels in the surrounding meiotic cytoplasm. Round spermatids (white arrows) possessed an enriched 'spot' of HP1α staining. **(d) **In *Cbx3*^*hypo*/*hypo *^testes, HP1α stained nearly all cells as in the wild-type. **(e) **Round spermatids (white arrows) were stained and possessed the typical nuclear focus of staining that was typical for this cell type. Bar = 100 μm. Click here for file

Additional file 9 **Figure s9**. Distribution and levels of HP1β staining in testes was not affected by the *Cbx3*^*hypo*/*hypo*^mutation. **(a) **Most cells within the wild-type testes were stained with HP1β-antibody. **(b) **A more detailed analysis of the HP1β staining showed that Sertoli cells (black arrowhead) were stained. HP1β did not stain meiotic chromosomes and was instead found in the meiotic cytoplasm (yellow arrows). There were also some cells at the periphery of the tubule, probably spermatogonia (black arrows), which were not stained. Inset shows pachytene spermatocytes with a punctuate pattern of staining (red arrows) probably heterochromatin. Round spermatids possessed an enriched 'spot' of HP1β staining (white arrows). **(c) **Expression of HP1β was lost at around the stage 10 spermatid stage. The box in **(c) **is magnified and two stage 10 spermatids are shown; one was still positive for HP1β (thick arrow, brown spermatid) the other was negative for HP1β (thin arrow; blue spermatid). **(d) **In *Cbx3*^*hypo*/*hypo *^testes, HP1β stained nearly all cells as in the wild-type. **(E) **In some tubules mature sperm (m) were visible. Bar = 100 μm. Click here for file

Additional file 10 **Figure s10**. There was no difference in number of tubules and gonocytes between wild-type and *Cbx3*^*hypo*/*hypo *^E17 embryonic testes. **(a) **HP1γ staining of E17 testes showed gonocytes heavily stained within the seminiferous tubules. **(b) **The HP1γ staining of *Cbx3*^*hypo*/*hypo *^E17 embryonic testes was very weak although the number of gonocytes was not significantly less than wild type (tubules are outlined with a hatched line). Bar = 100 μm. Click here for file
