# Correction: HP1gamma function is required for male germ cell survival and spermatogenesis

**DOI:** 10.1186/1756-8935-5-18

**Published:** 2012-11-21

**Authors:** Jeremy P Brown, Jörn Bullwinkel, Bettina Baron-Lühr, Mustafa Billur, Philipp Schneider, Heinz Winking, Prim B Singh

**Affiliations:** 1 Division of Immunoepigenetics, Department of Immunology and Cell Biology, Research Center Borstel, D-23845, Borstel, Germany; 2 Institut für Biologie, Universität Lübeck, D-23538, Lübeck, Germany

## Correction

After the publication of this work [1] it was brought to the authors’ attention that Figure three (Figure 1 here) contained a duplication error, where the HP1gamma staining for wild-type thymus and brain are identical. The correct figure is given below.

**Figure 1:**
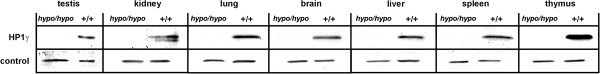
**HP1γ protein expression was dramatically reduced in *Cbx3***^***hypo*/*hypo ***^**tissues.** Protein expression was reduced to almost undetectable levels in testis, kidney, lung, brain, liver spleen and thymus tissues from the *Cbx3*^*hypo*/*hypo*^mice.
