# Supplementary figures and images for: HP1γ function is required for male germ cell survival and spermatogenesis
Source: Epigenetics Chromatin. 2010 Apr 27;3:9. doi: 10.1186/1756-8935-3-9 (PMC2877046; doi:10.1186/1756-8935-3-9)

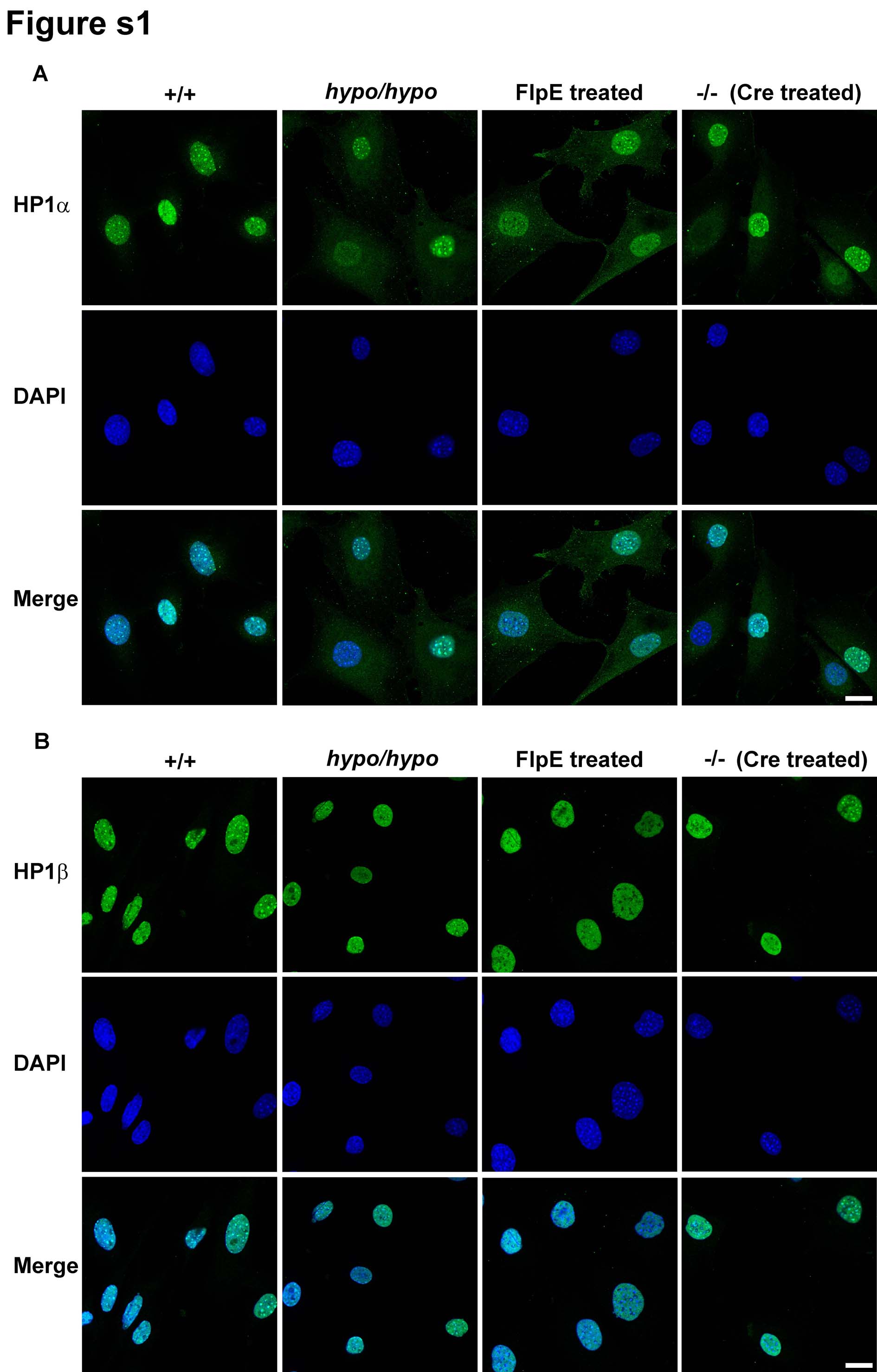

Supplement: Additional file 1 — Figure s1. Immunofluorescent staining of HP1α and HP1β was not changed in wild-type and Cbx3 mutant backgrounds. (a) There was no significant difference in HP1α staining on wild-type and Cbx3 mutant backgrounds (row 1, green). Row 2, staining with 4',6-diamidino-2-phenylindole (DAPI) of cells depicted in the panels above. Images of rows 1 and 2 were merged in row 3. (b) There was no significant difference in HP1β staining on wild-type and Cbx3 mutant backgrounds (row 1, green). Row 2, DAPI staining of cells depicted in the panels above. Images of rows 1 and 2 were merged in row 3. Bar = 20 μm. [file 1756-8935-3-9-S1.JPEG]

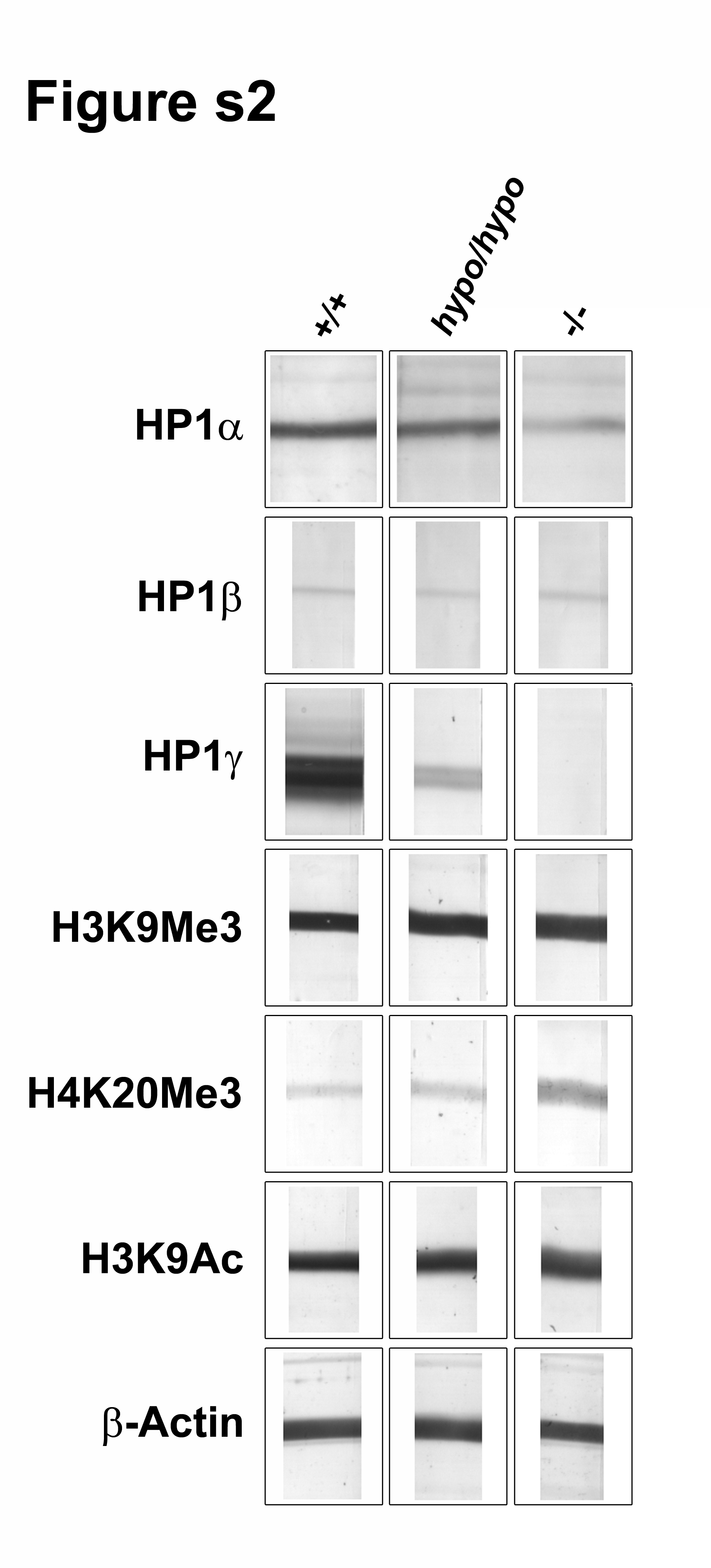

Supplement: Additional file 2 — Figure s2. There was no significant difference in the levels of HP1α, HP1β, H3K9ME3, H3K9AC in wild-type MEFs compared with Cbx3 mutant MEFs. There was a slight increase in H4K20ME3 levels in Cbx3-/- MEFs compared with wild-type and Cbx3hypo/hypo MEFs. [file 1756-8935-3-9-S2.JPEG]

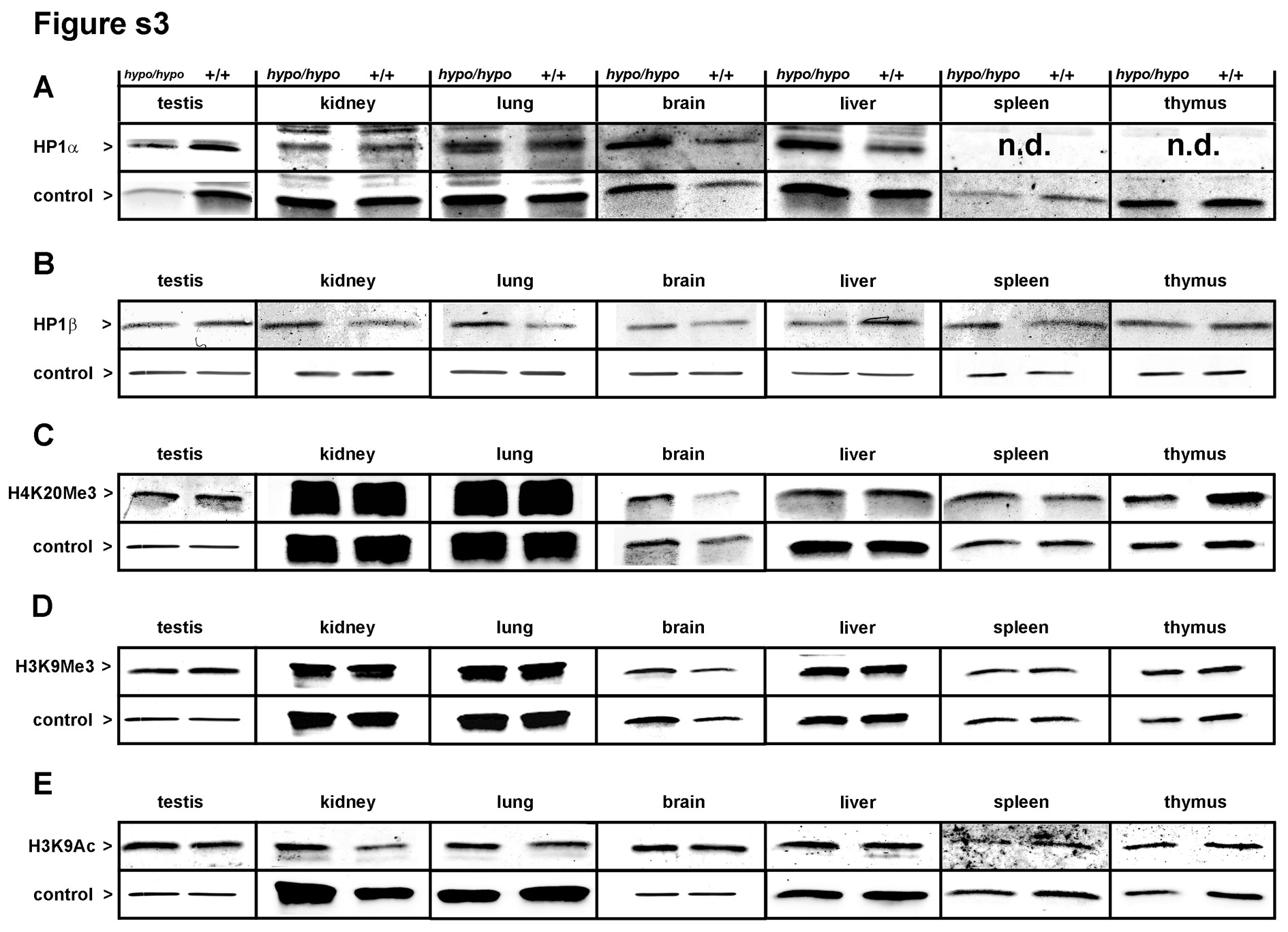

Supplement: Additional file 3 — Figure s3. There was no significant difference in the levels of HP1α, HP1β, H3K9ME3, H4K20ME3, H3K9AC in Cbx3hypo/hypo extracts taken from testis, kidney, lung, brain, liver, spleen and thymus tissues compared with extracts for the corresponding wild-type tissues. ND = not detectable. [file 1756-8935-3-9-S3.JPEG]

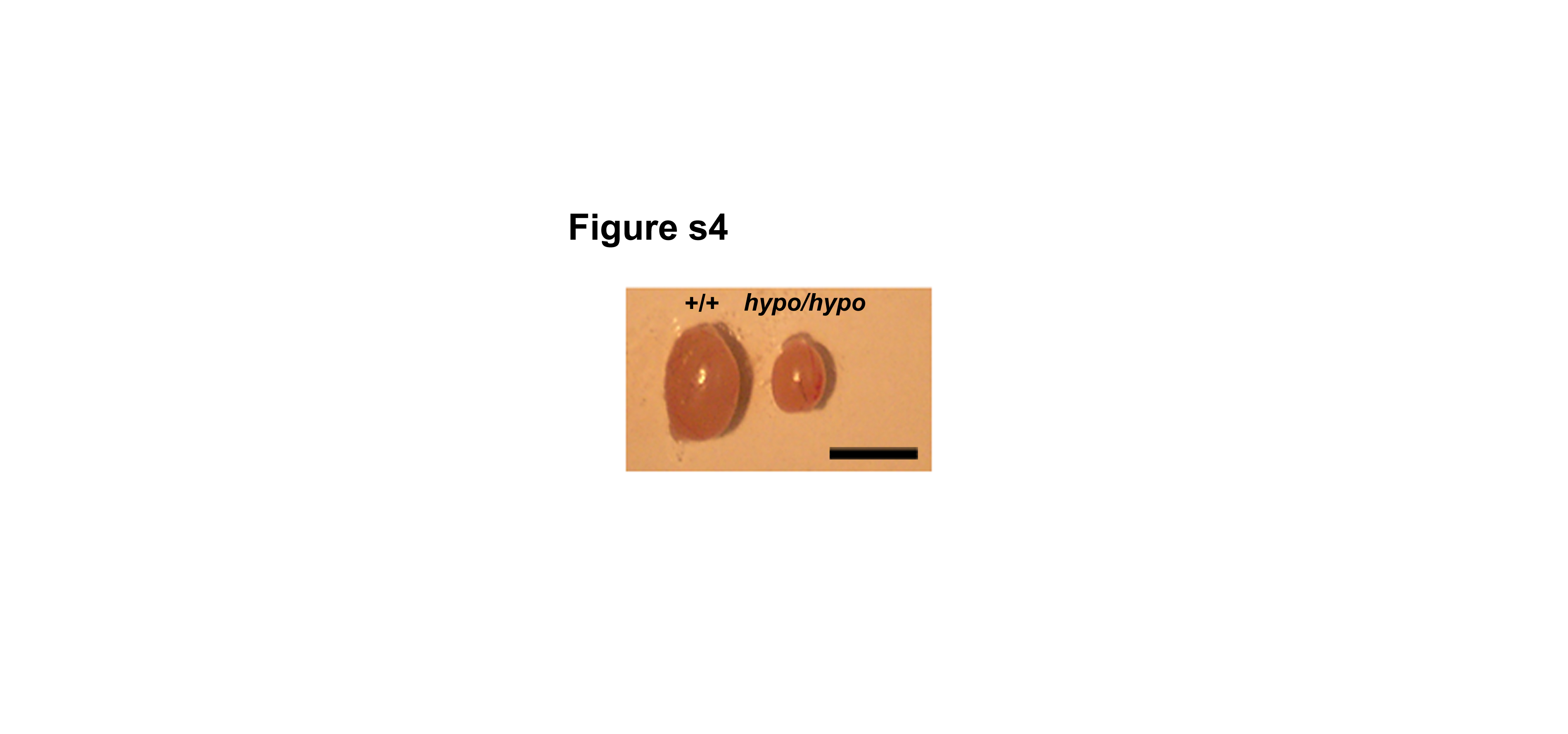

Supplement: Additional file 4 — Figure s4. Testes from Cbx3hypo/hypo males exhibited severe hypogonodism. Bar = 50 mm. [file 1756-8935-3-9-S4.JPEG]

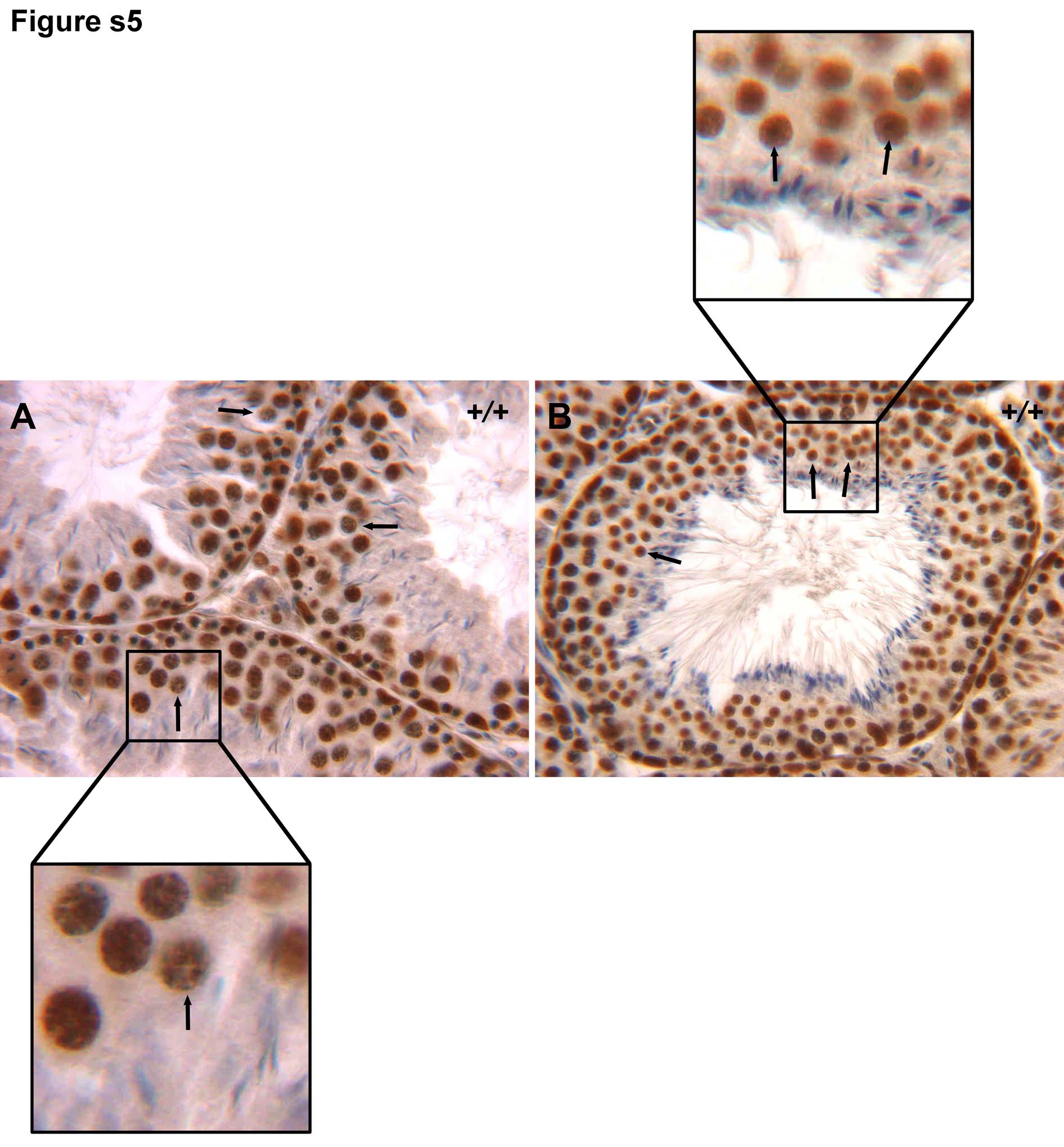

Supplement: Additional file 5 — Figure s5. (a) Many pachytene spermatocytes (black arrows) from wild-type animals had punctuate staining, with the intensely staining areas probably representing regions of heterochromatin (see inset). (b) Many round spermatids from wild-type animals exhibited a 'spot' that was enriched for HP1γ staining and probably represents the single block of heterochromatin that was characteristically found in these cells. [file 1756-8935-3-9-S5.JPEG]

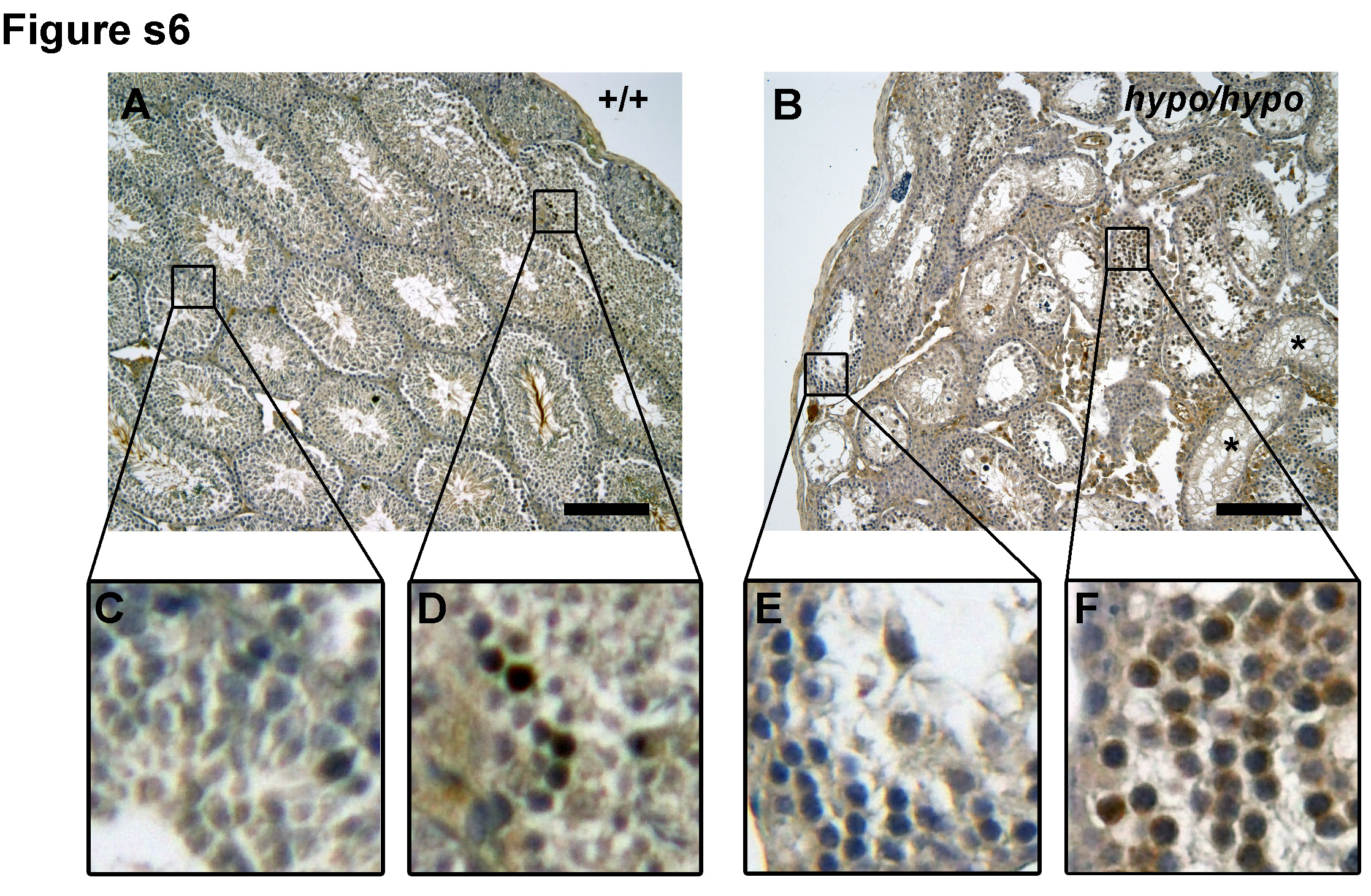

Supplement: Additional file 6 — Figure s6. LINE-1 (L1) retrotransposon expression was increased in Cbx3hypo/hypo compared with wild-type testes, as shown by an increase in germ cells staining positive for the L1-encoded ORF1 protein (ORF1p). (a) A wild-type testis section stained with the anti-L1-encoded ORF1p antibody, showing regions from two tubules, one tubule negative for ORF1p and the other tubule positive for ORF1p, which are magnified in (c) and (d), respectively. (b) A Cbx3hypo/hypo testis section stained with the anti-L1-encoded ORF1p antibody, showing regions from two tubules, one tubule negative for ORF1p and the other tubule positive for ORF1p, which are magnified in (e) and (f), respectively. The asterisks denote tubules that have the Sertoli cells-only phenotype and lack germ cells. (c) ORF1p-negative germ cells in a wild-type tubule. (d) ORF1p-positive germ cells in a wild-type tubule. (e) ORF1p-negative germ cells in a Cbx3hypo/hypo tubule. (f) ORF1p-positive germ cells in a Cbx3hypo/hypotubule. Bar = 200 μm. [file 1756-8935-3-9-S6.JPEG]

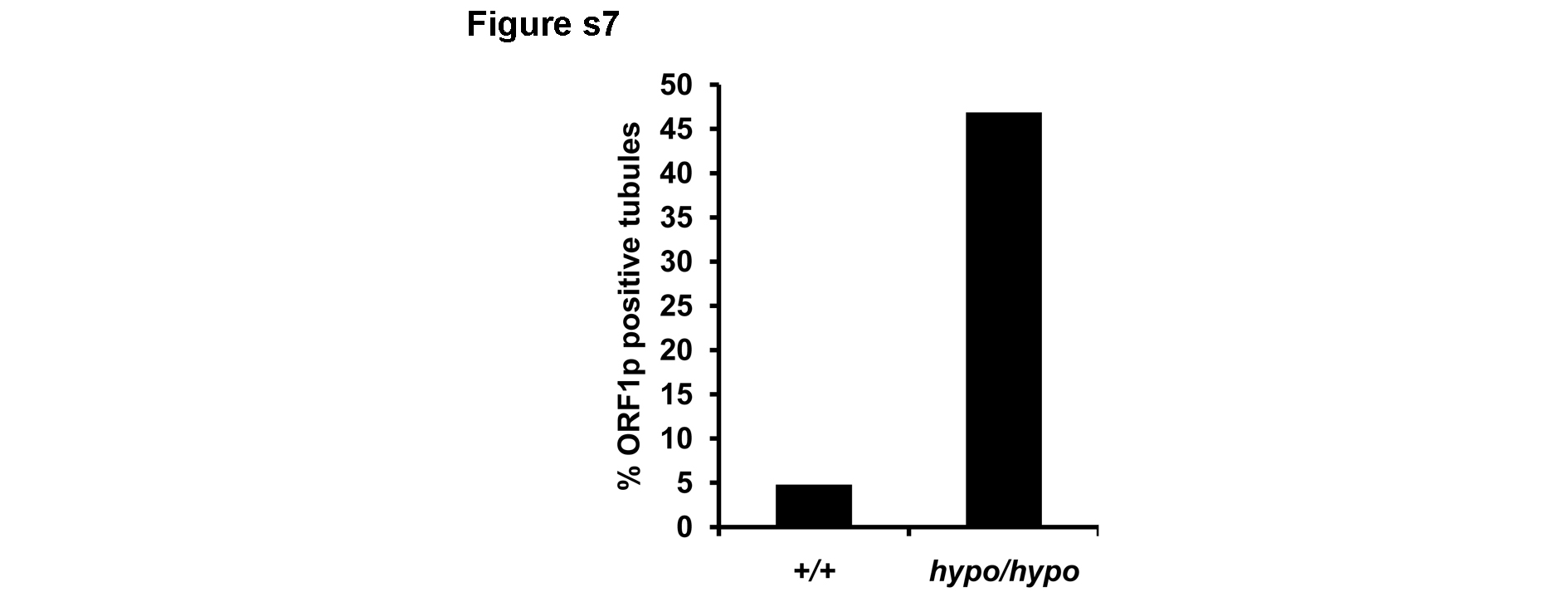

Supplement: Additional file 7 — Figure s7. In the Cbx3hypo/hypo testes section, 45% of the tubules contain ORF1p-positive germ cells compared with 5% of the tubules in the wild-type testes section. [file 1756-8935-3-9-S7.JPEG]

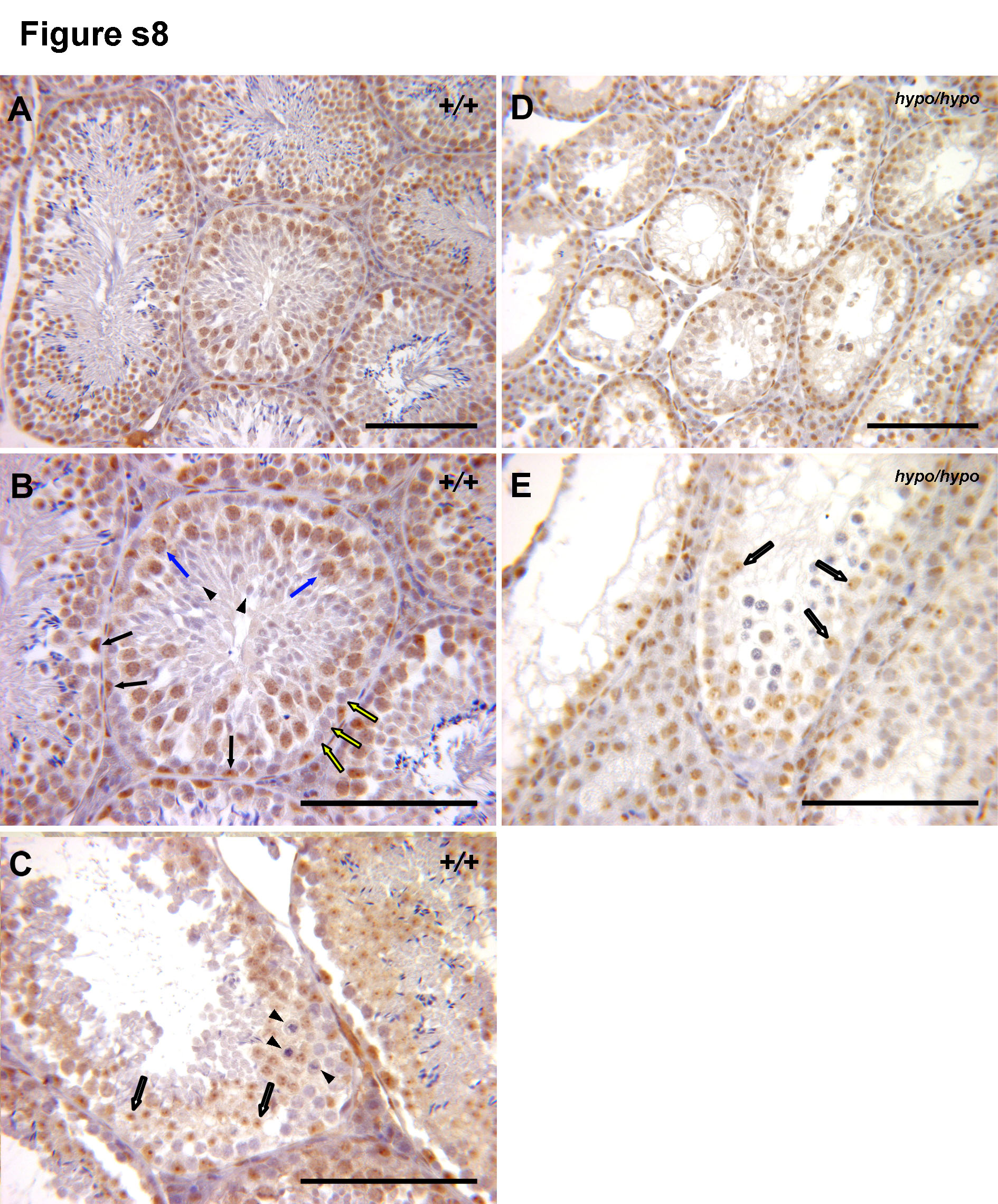

Supplement: Additional file 8 — Figure s8. Distribution and levels of HP1α staining in testes was not affected by the Cbx3hypo/hypo mutation. (a) Most cells within the wild-type testes were stained with HP1α antibody. (b) A more detailed analysis of the HP1α staining showed that Sertoli cells (black arrows) were clearly stained. Pachytene spermatocytes (blue arrows) exhibited punctuate staining, probably representing heterochromatin. HP1α staining was lost in stage 7 round spermatids (black arrowhead), which was earlier than for HP1γ (see Figure 4; Table 1). In contrast to the HP1γ staining, there were some cells at the periphery of the tubule, probably spermatogonia (yellow arrows), which were not stained. (c) HP1α does not stain meiotic chromosomes (black arrowheads) and was present at low levels in the surrounding meiotic cytoplasm. Round spermatids (white arrows) possessed an enriched 'spot' of HP1α staining. (d) In Cbx3hypo/hypo testes, HP1α stained nearly all cells as in the wild-type. (e) Round spermatids (white arrows) were stained and possessed the typical nuclear focus of staining that was typical for this cell type. Bar = 100 μm. [file 1756-8935-3-9-S8.JPEG]

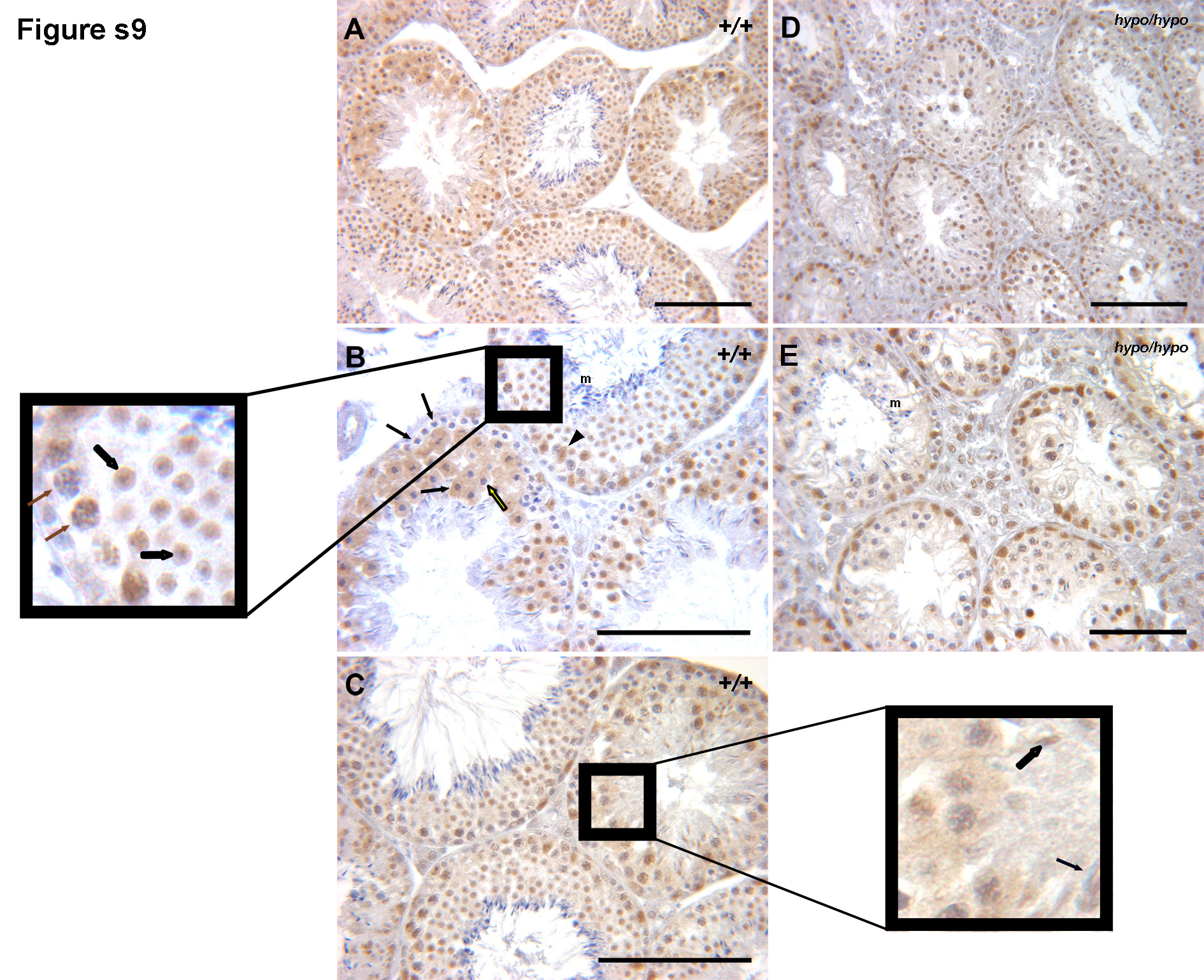

Supplement: Additional file 9 — Figure s9. Distribution and levels of HP1β staining in testes was not affected by the Cbx3hypo/hypomutation. (a) Most cells within the wild-type testes were stained with HP1β-antibody. (b) A more detailed analysis of the HP1β staining showed that Sertoli cells (black arrowhead) were stained. HP1β did not stain meiotic chromosomes and was instead found in the meiotic cytoplasm (yellow arrows). There were also some cells at the periphery of the tubule, probably spermatogonia (black arrows), which were not stained. Inset shows pachytene spermatocytes with a punctuate pattern of staining (red arrows) probably heterochromatin. Round spermatids possessed an enriched 'spot' of HP1β staining (white arrows). (c) Expression of HP1β was lost at around the stage 10 spermatid stage. The box in (c) is magnified and two stage 10 spermatids are shown; one was still positive for HP1β (thick arrow, brown spermatid) the other was negative for HP1β (thin arrow; blue spermatid). (d) In Cbx3hypo/hypo testes, HP1β stained nearly all cells as in the wild-type. (E) In some tubules mature sperm (m) were visible. Bar = 100 μm. [file 1756-8935-3-9-S9.JPEG]

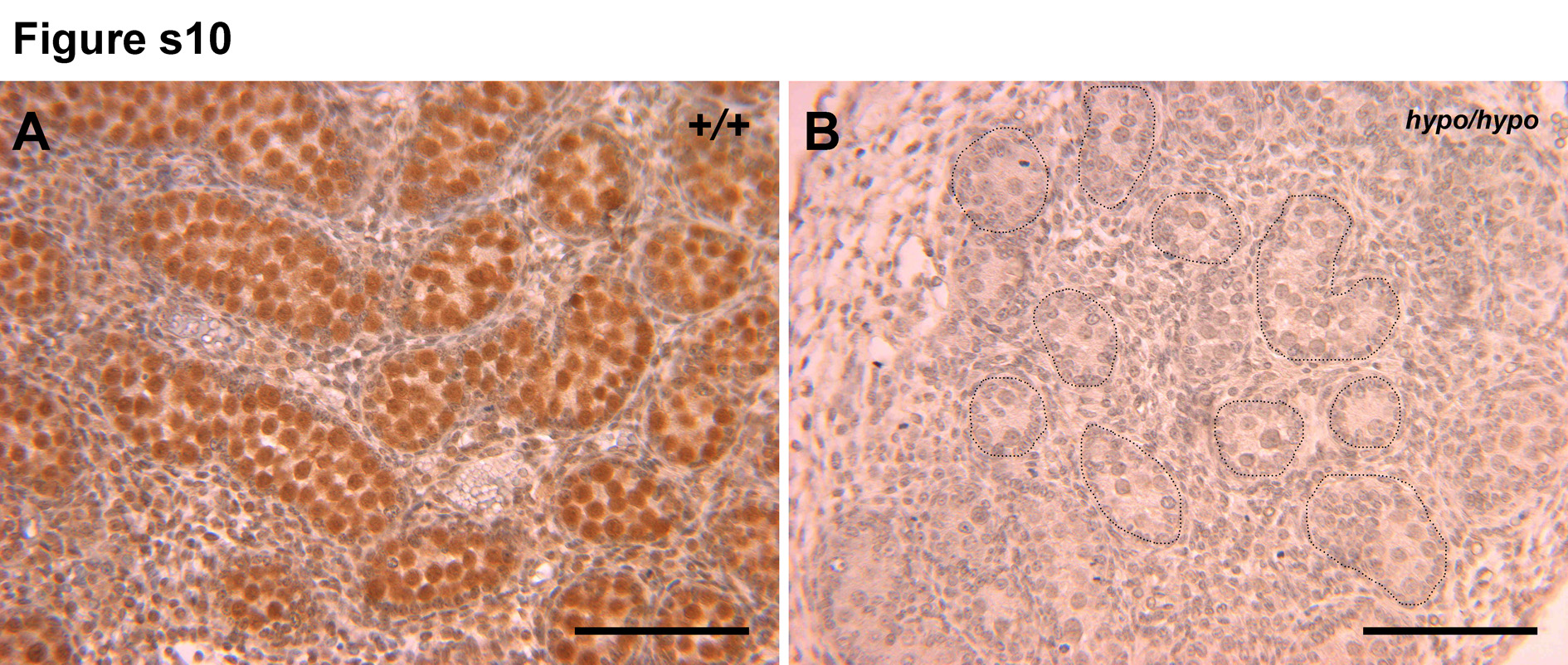

Supplement: Additional file 10 — Figure s10. There was no difference in number of tubules and gonocytes between wild-type and Cbx3hypo/hypo E17 embryonic testes. (a) HP1γ staining of E17 testes showed gonocytes heavily stained within the seminiferous tubules. (b) The HP1γ staining of Cbx3hypo/hypo E17 embryonic testes was very weak although the number of gonocytes was not significantly less than wild type (tubules are outlined with a hatched line). Bar = 100 μm. [file 1756-8935-3-9-S10.JPEG]
